# Supplementary material for: Lexical innovations are rarely passed on during one’s lifetime: Epidemiological perspectives on estimating the basic reproductive ratio of words
Source: PLoS One. 2024 Dec 5;19(12):e0312336. doi: 10.1371/journal.pone.0312336 (PMC11620389; doi:10.1371/journal.pone.0312336)
Supplement: Attachment — Submitted filename: Response to reviewers.pdf [file pone.0312336.s001.pdf]

Response letter (PONE-S-24-32537)

Dear editors and reviewers,

I would like to thank you for giving me the opportunity to revise the manuscript and for the huge number of suggestions for how to improve it. I have done my best to account for all of them. Some made me expand or rewrite parts of the introduction and limitations section. Some other comments required some more analyses, all of which improve the manuscript considerably, I think. In only a few cases, I needed to object; whenever this was the case I provide arguments below. The major points of improvement in the revision are the following:

- Limitations regarding **population structure** are discussed in more detail in 4.1 (R1 & editor)
- Sections 2.2.2 and 3. now feature a derivation and estimation of **margins of error** for all R-nought estimates (R1)
- Information about how corpora were **queried** was added to 2.1 (R1)
- Discussion and a brief robustness check regarding the use of **Google Ngrams** were added to 4.1 (R1)
- **Inflection points** of the growth curves were examined and juxtaposed with the range of birth dates of the survey participants to demonstrate that participants in the AoA surveys have largely witnessed the studied lexical changes; cf. Fig 5 (R1)
- The model description in 1.2 was expanded considerably to highlight the assumption of **homogeneous mixing** (R2 & editor)
- Discussion of **timescales** was added to 1.2 and 4.2 and backed up by literature (R2)

The code (https://gitlab.com/andreas.baumann/basic_reproductive_ratio) was updated as well and now contains all intermediate (preprocessed) data that are necessary to replicate the analysis.

In what follows, I provide detailed responses to each point made in the reviews.

Editor:

The two reviewers raise many questions, which you should try to take into account to all possible extent. In a few cases a bit more is asked for than is possible to deliver in terms of data available so the best response in these cases would be to briefly indicate the limitations and perhaps (again briefly) speculating on how results might be different given more information. One of such "what if" questions of my own relates to how things would look if we know more about differences in the amount of contacts among individuals. The estimated R0 looks pretty high for an average among all individuals, but one would expect that there is a non-normal distribution of the amount of contact individuals have when contacts include readers/listeners of printed and audio media, so an estimated mean might not be very meaningful. Perhaps the distribution of contacts is something like a power law, with the vast majority of individuals belonging to a fat tail contributing even less to the spread of innovations than the R0 = 6 would suggest. In whatever way this is done (just mentioning, speculating, trying to find data...), I think it needs to be taken into account to at least some minimal extent that lexical diffusion is different from the spread of diseases in that a single individual can reach a large part of a population in one instance.

The distribution of contacts is certainly important, and I have tried to discuss the impact of a skewed contact distribution in 4.1 ("It was shown that the invasion boundary in models that assume a more realistic population structure (scale-free networks) is lowered, so that R_0 estimates could be effectively underestimated (7,66)." etc.). I see that this was not made clear enough and I also didn't provide a suggestion for how the model could be improved in this regard. I now explicitly refer to mean-field models and data, which could be used to integrate a more realistic network structure, in 4.1. The assumption of heterogeneous mixing is now also highlighted in 1.2 (cf. comment by Reviewer 2).
Importantly, though, both linguistics and epidemiology are affected by this issue. The difference is, as pointed out correctly, that "a single individual can reach a large part of a population in one instance". I think, this is orthogonal to the question of how skewed the contact distribution is. Ultimately, it means that the parameter b in $\beta = bq\phi$ reflecting the number of contact events in which learning can take place (see section 1.2) is higher in the linguistic case than in epidemiology because linguistic transmission is not restricted to physical contacts (to be precise, thinking of vector-borne diseases or diseases that can be transmitted via fomites, like door handles, this restriction can be also relaxed in epidemiology; but it is much less common, I guess). The crucial point is: this must affect all words in a uniform manner. For that reason, it does not affect the ways of estimating R-nought *per se*. Section 1.2 now features a brief comment on this.

> There are a few typos etc.:
> l. 90: an average -> on average
> l. 91-92: perhaps move "not yet knowing the word" up to just after "contacts"
> l. 96-97: Heffernan et al. (2005) -> the paper of Heffernan et al. (23)
> l. 118: see van den Driessche (2017) -> see (24)
> l. 524: the lexicon growths -> the lexicon grows
> l. 530: data, mostly -> data mostly
> l. 615: delete "(Boberg 2004; Bailey 2004)"

Thank you, all fixed.

Reviewer 1:

> The paper proposes an interesting method transfer from epidemiology to linguistics, but suffers from simplification of language data and diachronic change factors to the point that I am not entirely sure it contributes much to linguistics in its current form, where the predictions are not tested yet rather strong claims about language change are made in the end. The unciritical usage of Google Ngrams for lexical data is also questionable. I would suggest the author to either: a) repurpose this as a method proposal paper, acknowledge the limitations, and remove the sweeping claims in the end; or b) figure out a way to actually test these predictions on a test set of lexical change cases, and work out how to estimate a confidence interval around the proposed R values and resulting claims. Don't get me wrong, it is an interesting idea, but the massive simplifications and ignorance of the nature of written corpus data will, I fear, only lead to backlash from linguists. Whereas this has potential to be actually useful, if presented carefully and tested appropriately.
>
> I will comment on a selection of passages below and attempt to explain my reasoning (the passages are copied from the pdf so line breaks and line numbers intersect with text, don't mind that).

Many thanks for the thorough review and the great set of suggestions! I have decided to do something in

between a) and b)). I have expanded the limitations section considerably and I address most of the issues that have been mentioned in this review (there are a few exceptions, which I will argue for below). More importantly, I now provide margins of error (i.e., confidence intervals) for all R-nought estimates. Two more remarks:

I wasn't quite sure what the suggestions of testing the predictions "on a test set of lexical change cases" means exactly, since the analysis covers about 2000 such cases for which R-nought is estimated based on acquisition, prevalence, and diachronic data. Perhaps this comment refers to fitting eqn (4) against *known* diachronic prevalence data (which is hard to obtain), but again, I am not entirely sure about that.

Second, thanks to this review, I have inspected the times when the respective spreading phenomena have occurred more closely and found that the majority of them are actually relatively recent and roughly match the range of ages of the AoA/prevalence-survey participants. This is good and hopefully mitigates some of the – certainly justified! – concerns regarding the mismatch between historical and contemporary data. See more on this below.

> After that, we will test predictions against diverse diachronic and synchronic linguistic data from four languages (English, Spanish, German, Italian; sections 2-3).
> --- This is my main problem with the paper, I don't see how the model is really "tested".

This is a very important point. The aim of this paper is not to test the model directly in the sense that trajectories are generated based on empirically estimated model parameters (beta, gamma, mu) and the trajectories being compared with empirically measured prevalence trajectories. While this could be done theoretically, it is hard to do this in practice: beta and gamma are not easy to estimate and getting historical prevalence estimates is difficult (if not impossible) as well (the same applies to epidemiological estimates of R-nought; see review by Heffernan et al. 2003). I have tried to make this clear in the first version of the manuscript (e.g., "In practical terms, however, it is not always easy to obtain reliable estimates for β and γ, and this more generally holds true for more complex models (23)." on p.6., "It is not at all easy to obtain word prevalence based on corpus data." on p.15 and the discussion following that). The crucial point is this: while desirable, we don't *need* to evaluate the model directly. Instead, we can test its predictions (namely that R-nought estimates derived in different ways match). This is what is done in the paper, and this is why the phrase "we will test predictions" is used throughout instead of "we will test the model". In any case, I have added a clarifying remark to the introduction.

> If there were, for instance, hypothetical political measures for banning a certain word, one would need to ensure that at least individuals are not able to adopt that word.
> --- This does not take into account uneven language policies and societal attitudes, e.g. in earlier centuries the upper and lower classes of these countries differed in their language use, and even today usage of certain words can be restricted to certain groups who may internally or externally regulate (think of racial slurs for example).

I totally agree. For that reason, I explicitly mention social structure when discussion herd immunity in section 4.2. I have added a note to section 1.2 to keep the reader informed.

> We test the predictions outlined above on the lexical level, i.e., by inspecting dynamics of words
> --- This is another problem I see, there is zero discussion on what counts as a word when it comes to

frequency data - is it any space-separated token in a corpus, a lemma, or something else? There is no discussion on lemmatization, but the word lists used are presumably lemmas, so were only the forms corresponding to dictionary forms counted? E.g. for English nouns it's the singular, so were plurals (analogously other forms of verbs) not counted in frequencies? English is morphologically simple, but e.g. German is already more complex with each lemma having a number of forms. How does that affect results?

Very good point! This information was totally missing in the manuscript. A detailed explanation was now added to 2.1 (in a nutshell, I have queried word-form frequencies, because there are separate AoA norms for different word forms).

words that have increased in frequency during the past two decades, and we will refer to such words as lexical innovations. We do so irrespective of whether these words have already been present initially at low frequency --- So which is it, innovations (not present before), or words that were low frequency and afterwards higher frequency?

It is the latter, exactly as defined in the text; it is not said in the text that innovations must not be present before. That is, some word could grow, e.g., because it got a new meaning.

Moreover, we only consider periphery words, which we define, somewhat simplistically, as words that are not used by everyone
--- I am not sure what this even means. The corpora used here have no data on speakers.

This must be a misconception. The corpora do not have any data on speakers, but the employed prevalence/AoA datasets, trivially, do. It is not claimed in the text that the used corpora contain speaker-level information (quite to the contrary, which is the whole point behind reconstructing prevalence trajectories in 2.2).

estimates for a large set of English from Kuperman et al. (42). Prevalence estimates for English were taken from Brysbaert et al
--- This is my other gripe - how are age of acquisition estimates of modern-day children supposed to say anything about (presumably mostly adult) lexical innovations of literate language users in 1820? What is the connection here?

This is a valid and important point that is addressed in section 4.1. Certainly, historical AoA estimates would be the preferred way of estimating the basic reproductive ratio based on diachronic growth for early innovations. There are ways of providing such estimates (cf. work by Bortaleanu et al. referred to in the text) but they come with their own problems.

Anyway, motivated by the reviewer's comment, I have had a look at the inflection points of the estimated logistic models: 50% of them lie within 1949 and 2008 (all languages; 1956 to 2008 if only English is considered), suggesting that many of the observed innovations are relatively recent phenomena. The majority of participants (69%) in the Kuperman et al. (2012) study were born in the 1970s and 1980s (total range 1929 to 1997). It seems, then, that the gap is less concerning than expected. In fact, in the case of English and German, birth dates even precede inflection points in a number of cases. I have added a note to 4.1 and the corresponding code to the RStudio project, as well as a new figure (Fig 5; see also below) that shows overlaps between the periods when the lexical changes happened and the period in which participants were born. I'd

like to thank the reviewer for raising this issue! I think that this immediately mitigates some of the other concerns put forth by the reviewer, particularly the remark about changing demographics.

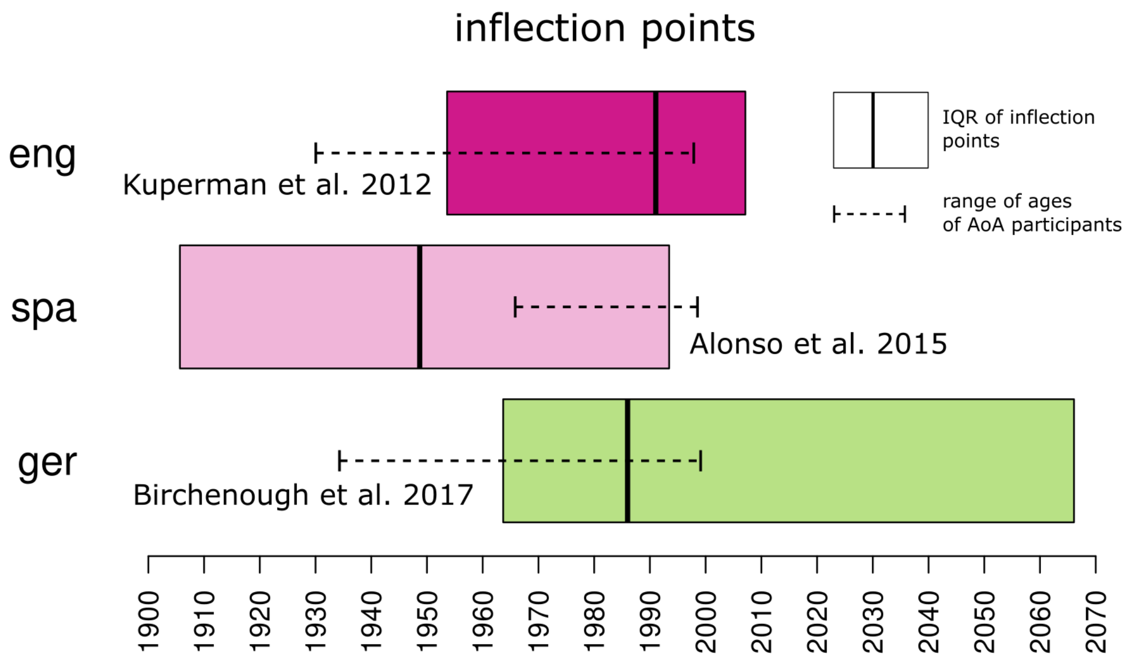

Note that the accuracy of the prevalence estimates substantially depends on sample size, i.e., on the number of participants they are based on. The respective sample sizes are about 300 for English, about 23 for German, about 50 for Spanish, and 25 for Italian<br>--- Am I to understand correctly that these sweeping claims about how lexical innovations work in human language are based not only on modern estimates but on a handful of e.g. 23 speaker optinions per language?

Yes, although I think that at least 300 informants per word for English (which seems to provide the most robust evidence in the present analysis, anyway) is actually quite a decent amount and far from a handful of speakers (again, this corresponds to a margin of error of at most about plus/minus 0.03 applying to the prevalence estimate). However, I do understand the concerns when it comes to Italian and German. Thanks to one of the reviewer's comments below, I now provide an error analysis, also considering the small sample sizes for these two languages. In fact, it turns out that the prevalence-based estimates of the basic reproductive ratio are not reliable for these two languages. I still decided to keep them in the analysis, since I do see a value in reporting things that do not work out well. This is now also acknowledged in 4.1.

For German, Spanish, and Italian, we needed to resort to normalized frequency trajectories extracted from Google books<br>--- Google Books Ngrams is a highly questionable source of language information - with some reservations it can be useful for e.g. grammatical things, but it has been shown to be questionable for lexical research purposes, as its sampling is pretty random and what may appear as "innovations" can easily be just sampling noise. Please see "Characterizing the Google Books Corpus: Strong Limits to Inferences of Socio-Cultural and Linguistic Evolution" by Pechenick et al and "The Impact of Lacking Metadata for the Measurement of Cultural and Linguistic Change Using the Google Ngram Data Sets—Reconstructing the

Composition of the German Corpus in Times of WWII" by Koplenig, and consider if this is a valid corpus for your study.

Very good point! I have added to things: first, I acknowledge the problem and some of the literature kindly provided by the reviewer in 4.1 (when discussing the range of inflection points; and in a new paragraph dedicated to the issue). Second, I conducted a robustness check which compares COHA estimates against estimates from Google Books for a sample of 250 words. About 79% of all words in this sample show strongly and significantly correlating frequency values in COHA and Google Books (measured in terms of Pearson's r of the two trajectories; this was done for each of the 250 words). About 20% do not show a significant correlation (see figure below; not shown in the manuscript). A small fraction of 1.6% show a negative relationship. It seems that, extrapolating from English, working with Google Books in this specific study would not be very harmful.

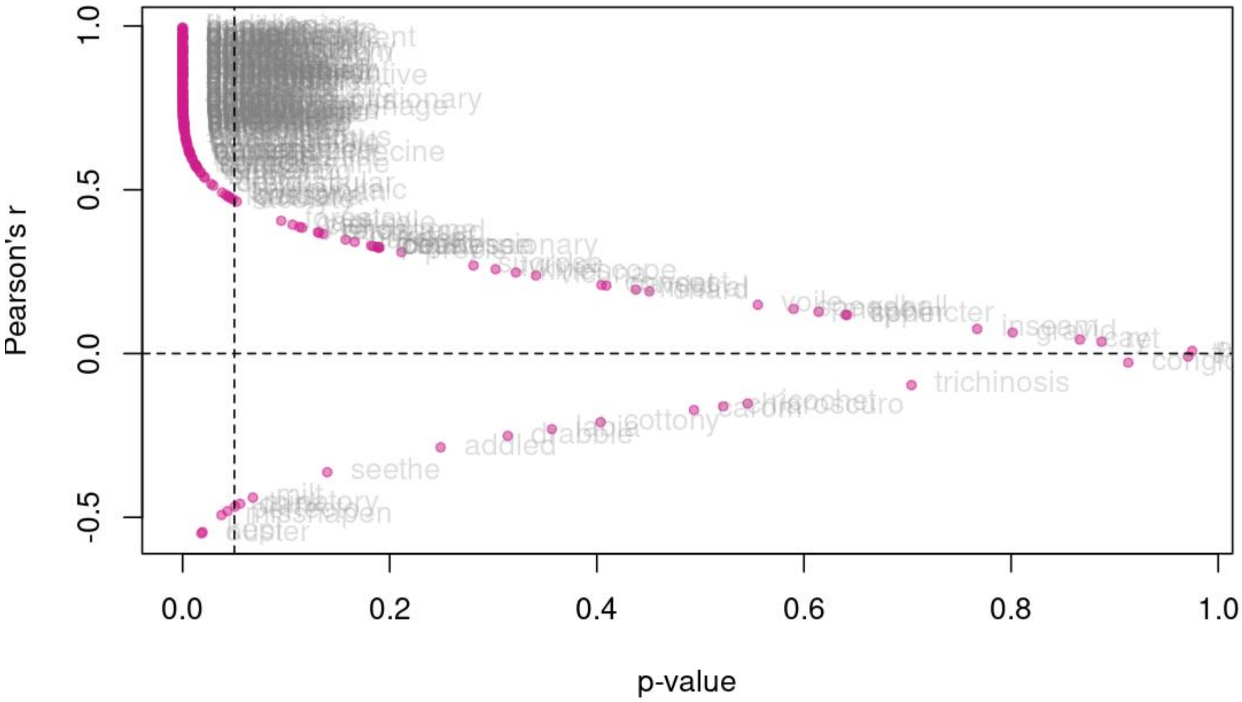

Also, think for a moment about the speaker communities. You are comparing US English where the speaker population went from around 8M to 300M during the COHA corpus period; Italian which is also spoken mostly in one country, German spoken in 4+ countries, and Spanish which by now has half a billion native speakers and is the official language in 20+ countries (in varieties which the Google Books ngram data does not differentiate between). How are these even remotely comparable in a model that relies so much on the individual speaker, and how can such a model reliably applied to such volatile and heterogenous populations?

I can hardly argue against the observation that the examined populations differ substantially from each other. The four speaker populations are characterized by very different social and geo-political patterns. I want to make three points, though:

First, I do not see why the fact that the model relies on individual speaker interactions is a problem here. I do understand that realistic social/geo-political patterns should be ideally integrated into the model. I now emphasize this in the limitations section; crucially, compartment models are flexible enough to do so. The epidemiological literature provides numerous examples for how this can be done, and much of the social/geo-political structures explored through compartment models there are, I think, useful in linguistic models as well (see, e.g., Silal et al. 2023 for a sophisticated model of COVID dynamics in South Africa).

Second, yes, social/geo-political patterns will affect how words spread. One thought on this: it could be that there are differences between estimated R-nought values are actually a reflex of the differences between the respective speaker populations. A speculative example: the lower R-nought for Spanish based on growth data could be an effect of the globally more scattered geographic distribution of that language. Evidently, testing such claims require data from more languages than just four. I do not want to expand on this in the manuscript, but I have added a brief note to the first paragraph in 4.2.

Third, regarding increasing population size: yes, very good point. There is analytical work by May and Anderson (1985) showing that the age-of-first-infection approximation still holds for expanding populations. The only difference is that LE is then replaced by the reciprocal of birth rate. Note that according to the formulation of the model in the manuscript, the reciprocal of birth rate is (almost) the same as LE, since dying individuals are immediately added to the non-user class. But yes, using birth-rate estimates instead of LE would have been an option. I have added a comment on this in 4.1.

> We use the collected age-of-acquisition estimates, assume a life expectancy of 75 years (https://data.worldbank.org/), and estimate the basic reproductive ratio as (i) 0= /. Note that life expectancy has increased through the past two centuries and that women have, on average, a higher life expectation than men, so that the assumption of a single point estimate is clearly simplistic.
> --- Life expectancy in 1850 in the US was around 40 years, almost half your assumption. I don't see why this simplification is made, instead of working known expectancy estimates into your model.

This is a good point that, I think, is mitigated by the (new) observation that most of the inspected innovations are relatively recent and match with the range birth dates of the participants in the AoA surveys (see response above). That is, the average of about 75 does not seem to be too bad. I now report the margin of error of LE (which is about 1) and consider it in the error analysis (2.2.2). The simplification of having a single point estimate of LE is made since the implementation of a dynamic LE would make the model and the derivation of R-nought analytically much more complex.

> In the UK, for instance, life expectancy has increased from about 71 in the early 1960s to about 80 in the 2000s (Fig 3, left). Notably, the UK displays the smallest increase in life expectancy.
> --- The COHA is US American English. I don't see how the UK is relevant here.

I have now merged data from the UK and the US since the prevalence dataset (Brysbaert et al. 2019) also involves speakers from both countries. See 2.2.1.

> It is not at all easy to obtain word prevalence based on corpus data. This is because corpora often feature only a small number of authors and because the amount of text per author is likely not representative of their linguistic knowledge (let alone the fact that the author population in linguistic corpora is not necessarily representative of the whole population

> --- This is a massive problem on its own, acknowledged but not taken into account - but the related problem is differential number of speakers over time - more people simply became literate over 200 years in these countries, so if the corpus is randomly sampled, the number of speakers in it should be significantly different, and ignoring this surely introduces bias in a model so heavily reliant on the unit of individual.

I am not 100% sure whether I have understood the criticism correctly. I do *not* use prevalence estimates based on authorship. Instead, prevalence trajectories are reconstructed based on contemporary prevalence estimates and frequency data derived from corpora. Or is the criticism that frequency trajectories in diachronic corpora are not reliable *per se* because of the biased set of authors? It's a valid point but this would apply to literally all frequency-based corpus studies. Perhaps, this point is mitigated by the analysis shown in Fig 5 anyway.

> Just a note on 3D plots, and tables and figures with abbreviations - these are just very hard to read. Would be much easier if the terms were written out on the axes and in tables. 3D plots like these are almost unreadable; could just use color for the 3rd dimension where needed.

I have replaced 3D plots by 2D plots with separate panels for the stability dimension.

> The analysis of expected values for the basic reproductive ratio (for all languages and methods) shows that estimates typically range from about 3 to about 14
> --- Here's my third main issue with the paper: what do we learn from this, and how do we know if these numbers are anywhere near true values? There is some post-hoc discussion later, but what use is this number for linguistics and advancing our understanding of how language works and changes?
>
> Given the limitations discussed in the previous section, does the approach adopted here represent an accurate model of lexical spread? Certainly not – as it depends on simplistic assumptions. If the model assumptions are met, however, our analysis suggests that periphery words, on average, show a basic reproductive ratio of about 0 ≅ 6, with inter-quartile ranges going from about 2 to 17, depending on the language and estimation method. This puts spreading periphery words – in certain settings – roughly on par with infectious diseases like Rubella
> --- Again, rhetorically, so what? The number that may or may not be correct is like a number if a completely unrelated domain. What is the confidence/margin of error here, given all the (considerable) limitations here pertaining to corpus data, population data and the model, are we talking 6±1 or 6±6?
>
> Finally, the estimate 0 ≅ 6 also tells us about the role that individuals play in the propagation of new words through contact networks. Mean personal network size was shown to equal about 600 (80). This 573 reflects all individuals that one gets to know during one's life time. This means that a single individual that picks up a lexical innovation passes it on to only about 1% of their personal contacts – notably,
> --- Exactly the same critique as above.

This is an excellent point. Thanks for raising this issue. I have now invested some efforts into deriving margins of error for all R-nought estimates. For estimates based on acquisition and prevalence, this works in a very straight forward way. I use error propagation based on the first-order Taylor expansion of the respective formulas. Margins of error of life expectation, age of acquisition and prevalence can be obtained relatively easily from demographic data and employed AoA/prevalence datasets. Unfortunately, getting a margin of error for the growth-based estimate required a little more work since growth rates are not defined by a closed

formula and since trajectories depend on prevalence estimates. I decided to bootstrap the 95% confidence interval for each growth rate (to be used in the error propagation). I am aware that the bootstrapping procedure is not ideal since it ignores the fact that token frequencies are also point estimates (a thing that is in fact overlooked very often in corpus studies). Long story short, for English and Spanish it is about 6±2 (cf. figure below, now Fig 8 in the manuscript).

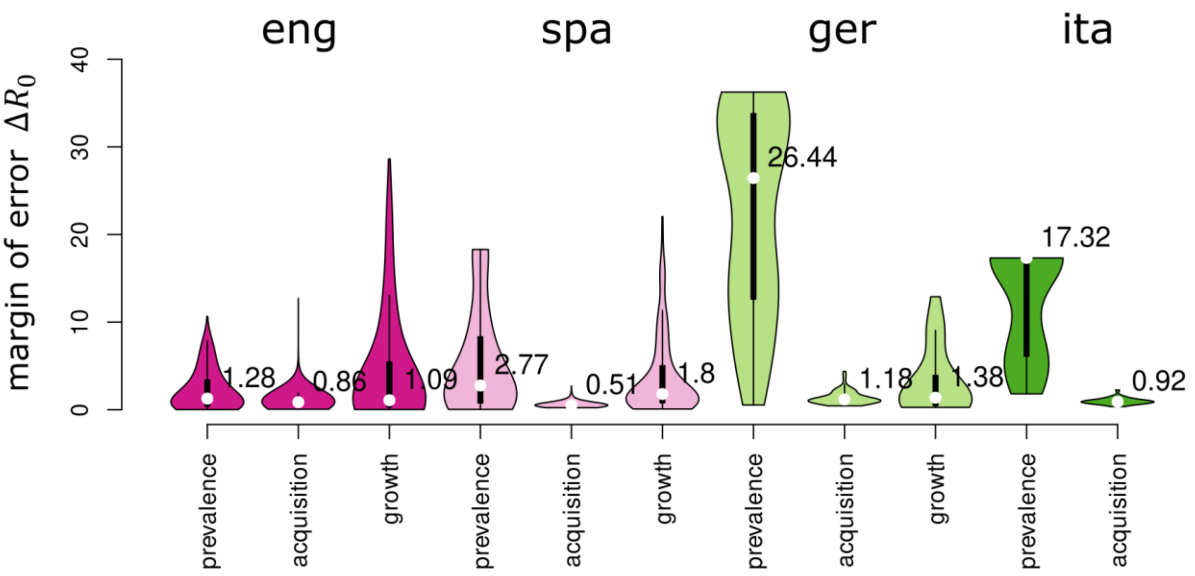

As to "but what use is this number for linguistics and advancing our understanding of how language works and changes?": we learn from this analysis that when I acquire some emerging (!) word like 'hipster', then roughly 6 people will learn it from me during my whole lifetime. It provides insights into how much each individual contributes to the spread of some new word (just as its epidemiological cousin informs us about how much each individual contributes, on average, to the spread of a disease). It is an average score, sure, and there will always be individuals that are more or less impactful in this respect, but so are average measures of personal network size or the average amount of text/speech that an individual produces per day. Each of these pieces of information is, I think, relevant for our understanding of how language (change) works.

> throughout the whole period during which that individual knows and uses that word. If one considers an average offspring count of about 1 (per individual),
> --- This is below rate of replacement, and well below the average number of children historically. Making this assumption equates to assuming these four languages or their speaker populations have gone extinct by now.

This is not correct. If each individual (NB: *not* each reproductive woman) is replaced by one offspring, then population size stays roughly constant (I know, it is slightly more than 1 because of child mortality, but this is not the point).

> As a final comment, if such a model would be tested against known case, I expect there would need to be some way to account for an error term or variance left undescribed. These current estimates assume a singular process of lexical innovation, whereas language change is complex and affected by numerous factors mostly or entirely ignored here. To provide some ideas:

- Age and mixing is briefly discussed in the end, but nothing about sociolinguistic prestige (tons of literature on that)
- Selection and drift literature is cited but it seems like drift could be one way to conceptualize the residual/error not described by this model (for a more recent update since the Newberry paper cited here, see e.g. "Reliable identification of selection mechanisms in language change").
- Borrowings are mentioned, but how the proportion of L2 speakers affects language is ignored (see e.g. "Larger communities create more systematic languages", "Language Structure Is Partly Determined by Social Structure")
- Stadler et al is cited but this potential mechanism is ignored; relatedly, other works have looked into neighborhood/topical effects ("Quantifying the dynamics of topical fluctuations in language" "Where New Words Are Born: Distributional Semantic Analysis of Neologisms and Their Semantic Neighborhoods")
- The issue of semantic change is practically ignored - it is not just new forms being innovated, but new meanings arise and old words are given new meanings (maybe useful: "Algorithms in the historical emergence of word senses", "Survey of Computational Approaches to Lexical Semantic Change")

I am not saying a model would need to necessarily include parameters for all these things, but some acknowledgement that it is not just neutral disease-like passing of new words would be prudent, and if the model were to be evaluated on some test set, it would make sense to reserve an error term or otherwise account for the fact that there are other things happening in language change.

This is a great set of suggestions. One general remark: unless for modeling drift (see comment below) integrating a general error term that accounts for 'all what's left' is, at least according to my knowledge of the ODE/dynamical-systems literature, very uncommon. It is clear that all of the parameters reflect expected values. There might be other variables that drive the growth of a word/pathogen (and very likely so); but variables are usually explicitly implemented into the system of differential equations at hand. I am very hesitant regarding this suggestion. That is, if one integrates drift/stochasticity it needs to reflect a specific mechanism, and one needs data to measure it.

More detailed comments on the specific suggestions:

Social structure is now emphasized more strongly in section 4. Prestige is an interesting point. I have recently done some work on a version of the model that also features socio-pragmatic conformity and non-conformity biases (Baumann et al. 2023); not exactly the same, but a related mechanism. I now refer to this in 4.2. However, as relevant as these mechanisms might be, I do not think that they can be reasonably modeled by an error term.

Drift: It is a wonderful suggestion. In fact, I had parametric drift in an earlier version of the manuscript, with drift estimated from the fluctuations of the frequency trajectories. In Baumann (2018) I have already had a brief look at the stochastic version of the model (with parametric drift to reflect variability in transmission rates) and I originally planned to provide some more empirical assessment of that. I decided to drop this part, though, because (a) it would have required some more extensive derivations of how R-nought can be estimated, since I am (b) still fiddling around with the proper data to use (see also comment on semantic fluctuations below), and because (c) the manuscript was already long anyway. I am still working on this. Thanks for the reference, in any case! I now briefly discuss the issue in 4.1.

Proportion of L2 speakers: I am aware of this, but I ignore the mechanism for the following reason: it is hard to tell whether it only affects (a) how often new words are imported into a language (very plausible) or also (b) how easily the word is transmitted within the speaker population once it is there (a bit less clear). If it is only (a) then R-nought *per se* will not be affected by the fraction of L2 speakers. If it is also (b) then growth rates could be higher for exoteric languages. Again, definitely an interesting question (see response above about socio/geo-political structure), but it requires more than four languages to address it.

Topical drift and semantic relationships: I agree, this is relevant for lexical acquisition and change. I now explicitly mention the assumption that words evolve independently in the limitations section (4.2), together with references to related literature (the paper by Ryskina et al. 2020 is great and I wasn't aware of it – thanks for sharing this!). However, I am skeptical as to whether a general error term would be the best way to account for such interactions. It would be better, in my eyes, to analyze this in terms of a multidimensional population model in which multiple words (or word variants) can compete against (or support) each other. Interaction rates could then be estimated based on semantic similarity/cooccurrence rates. I am very sympathetic with respect to such kind of modeling, but I believe this goes way beyond the scope of this paper. A stochastic (drift) term is, I think, only warranted if one wants to model that lexical transmission is affected by semantic fluctuations that need to be measured in some way (as in Cassani et al. 2020, and some recent research of our own, Baumann et al. 2024).

A note on the criticism "The issue of semantic change is practically ignored": it is not. It is only kept abstract in that I look at words that grow for whatever reason (cf. "We do so irrespective of whether these words have already been present initially at low frequency, whether they are associated with new concepts or are involved in onomasiological competition, whether they are new word formations or have been adopted from a different language."). To make it clear, I do not at all argue against lexical transmission, i.e., the parameter beta in the model, being affected by semantics (e.g., properties of the semantic neighborhood of a word or semantic features like a word's concreteness, cf. Snefjella et al. 2019). But it is just one of many determinants of beta. Some words will have semantic properties that promote propagation, others will be propagated less easily due to some semantic properties; both is implicitly reflected in the basic reproductive ratio and leads to higher and lower R-nought values, respectively. For the same reason, I also don't model, say, phonological properties of words that influence transmission.

Reviewer 2:

> In this work, the author views lexical dynamics as an infection process, whereby a particular lexical item can infect a user and thus spread through a population. In particular they focus on computing the basic reproduction number R0 for four different languages through three different methods. Subject to underlying assumptions on the dynamics being correct, all three methods should give similar results and the author finds that they do indeed correlate well, with a median value (over words in language) of around 6. The author performs a careful statistical study of the results, showing that the correlations in particular, and the findings in general, are robust. The discussion section does a good job of examining the implications of the results for language dynamics, and in addressing the limitations of the study.

Thank you!

> Overall I have little to criticise, although I think the manuscript could benefit from a little more discussion of the following points:

1. The comment that "most average R0 estimates fall into a similar range, across languages ... is reassuring ... and potentially more revealing" deserves expansion. The easiest R0 to intuit is the the one obtained by dividing life expectancy by age-of-acquisition. A value of 6 is therefore indicative of the fact that the median word is typically acquired at around 1/6th of one's overall lifetime, which seems broadly reasonable even given historical changes in life expectancy. The question then is: how much scope is there for variation in values of R0 given how they are defined? If the answer is 'not much' then we should perhaps not be too surprised that we get similar ballpark figures back. (I might argue that the correlations between values from the different methods are therefore more revealing, because there it's less obvious that the variables that they depend on should be related.)

Yes, I think it is not so surprising that R-nought estimates derived with the same method/data (e.g., AoA) are relatively close to each other (also because AoA estimates in the surveys do not go beyond 20 years). However, what *is* potentially surprising is that estimates derived with three different methods and based on three different data resources roughly fall into similar ranges. Of course, according to the model they should; but given all the simplifying assumptions of the model and limitations when it comes to the data it is interesting (and good) to see that we still get such similar ranges. This is what I meant in the manuscript; I have added a clarifying remark (4.1).

2. Following from point 1, it would help to flag up the basic assumptions behind the model defined by the equation on p8. (NB: it is very helpful to number equations, as it makes it easier for readers to refer to them.) Key among these are the chosen number of compartments, and the fact that populations are assumed to be well-mixed. These assumptions are discussed towards the end of the manuscript, but I think it is worthwhile to emphasise that the different ways one can define R0 are a consequence of these assumptions. Thus if one does not get similar values from the different methods, one possible explanation is that the underlying assumptions are not valid.

I agree, I just realized that heterogeneous mixing was not even mentioned in 1.2 (only 1.1). This is now fixed. The paragraph on the assumption of heterogeneous mixing (vs. a more realistic network structure) in 4.1 was slightly expanded (see also comment by the editor above that goes into the same direction). Equation numbers were added.

3. Related to point 2, it would help to clarify what the infection event that causes a transition from the N to U compartments is envisaged to be and what the characteristic timescale of infection is. Within the 'age of acquisition' approach, it seems implicitly the case that N to U event is a childhood language acquisition event, which one could potentially question for lexical dynamics. For example, when there is an innovation, a word could also spread through an adult population without having to wait to be learnt by the next generation of children. I think this impacts on the discussion around the relatively small number of people that we infect with our lexicon: if we have only childhood language acquisition in mind, then 6 is actually quite a big number, since most individuals tend to act as a caregiver to a small number of children. The timescale question meanwhile I think is important when making comparisons to diseases like Rubella and Covid-19. R0 doesn't say anything about timescales - just how many people you pass the infection onto, on average - but it is clear from these comparisons that the despite having different R0, the timescale over which a lexical item infects a population is much longer than that of an infectious disease. It is not immediately obvious to me what sets the timescale in the linguistic case, especially if infection can happen

through use as well as acquisition. It would be interesting if the authors could add any insights they have on this issue to the manuscript.

The point about acquisition is very important. Although AoA is used to infer R-nought, there is no implicit or explicit assumption that transmission only takes place during childhood. The narrow age range is rather a limitation imposed by the employed datasets (as a matter of fact, individual subjective AoA estimates beyond 25 years are sometimes excluded in these studies to prevent outliers that could skew (mean) AoA norms). The model does not make any difference between age groups and hence allows for interactions among adults as well. This is now emphasized in 1.2. in the extended version of section 1.2 it should be now also made clear that the "infection event" corresponds to any kind of linguistic interactions among individuals.

As to timescales: This is a very interesting question. I think the answer is hidden somewhere in section 4.2, more specifically: "the prolific, i.e., infectious, period of words is much longer than that of the above-mentioned infectious diseases". It is absolutely correct that R-nought does not tell us anything about timescales, i.e., there is no one-to-one relationship between R-nought and rate of growth (viz. an increase of x individuals per time step). Diseases like Rubella and Covid-19 spread faster than words (we observe a higher growth rate r) but their infectious period L is much shorter. Hence, the approximation $R_0 \cong 1 + r \cdot L$ can yield similar R-nought values (here: about 6) for both, words and diseases. Once acquired, words simply have more time to be passed on to someone else than pathogens that face an immune response that ousts them over a period of a couple of days or weeks. I have added some thoughts on this to 4.2 and now say in the introduction that R-nought is a dimensionless quantity to sort out any potential misunderstandings early. In addition, I have added some more discussion about the prolific duration $L = LE - AoA$ and some evidence for why it makes sense to assume that words are not easily abandoned once acquired (in particular, Brysbaert et al. 2016 show that vocabulary size increases at least up to the age of 70 years).

Additional references:

- Baumann, A., Stephan, A., & Roth, B. (2023, December). Seeing through the mess: evolutionary dynamics of lexical polysemy. In *Proceedings of the 2023 conference on empirical methods in natural language processing* (pp. 8745-8762).
- Baumann, A., Scheicher, A., Böhm, I., & Hartmann, S. (2024). Aspects of semantic change and how they interact with lexical acquisition. In *Proceedings of the Annual Meeting of the Cognitive Science Society* (Vol. 46).
- Cassani, G., Bianchi, F., & Marelli, M. (2020, September). Words that shift are learned later: A study on the relation between diachronic semantic change and age of acquisition. In *Architectures and Mechanisms for Language Processing*.
- May, R. M., & Anderson, R. M. (1985). Endemic infections in growing populations. *Mathematical Biosciences*, *77*(1-2), 141-156.
- Silal, S. P., Pulliam, J. R., Meyer-Rath, G., Jamieson, L., Nichols, B. E., Norman, J., ... & Moultrie, H. (2023). The National COVID-19 Epi Model (NCEM): Estimating cases, admissions and deaths for the first wave of COVID-19 in South Africa. PLOS Global Public Health, 3(4), e0001070.
- Snefjella, B., Généreux, M., & Kuperman, V. (2019). Historical evolution of concrete and abstract language revisited. *Behavior research methods*, *51*, 1693-1705.
